# Supplementary material for: OCT4 enhances the firing efficiency of late DNA replication origins in mouse embryonic stem cells
Source: Nat Commun. 2026 Jan 15;17:1686. doi: 10.1038/s41467-026-68389-1 (PMC12910074; doi:10.1038/s41467-026-68389-1)
Supplement: Supplementary file 1 — Supplementary information [file 41467_2026_68389_MOESM1_ESM.pdf]

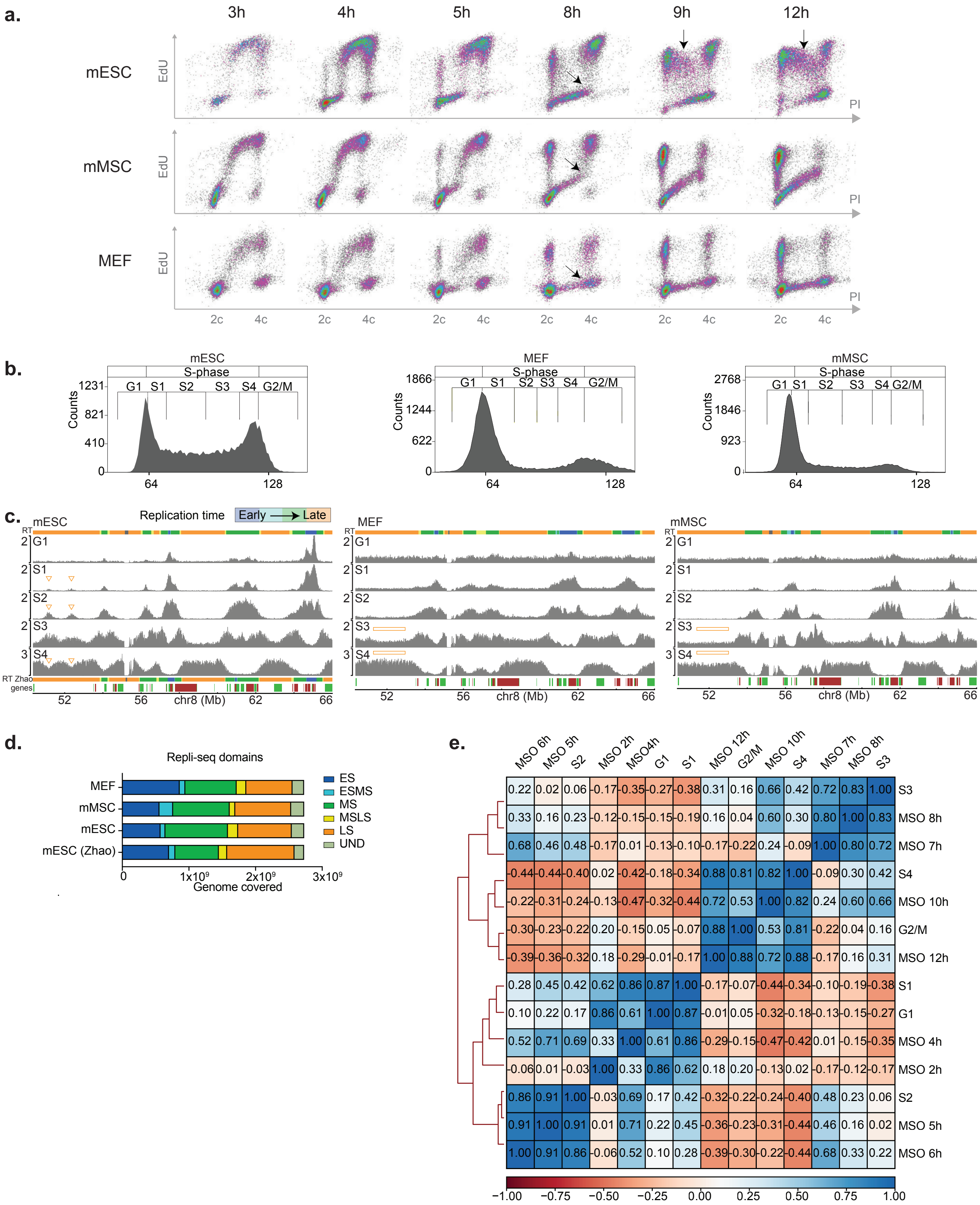

a.
3h
4h
5h
8h
9h
12h
mESC
mMSC
MEF
EdU
PI
2c
4c
b.
mESC
MEF
mMSC
S-phase
G1
S1
S2
S3
S4
G2/M
Counts
1231
821
410
0
1866
1244
622
2768
1846
923
64
128
c.
Replication time
Early
Late
RT
RT Zhao
genes
chr8 (Mb)
52
56
62
66
d.
Repli-seq domains
MEF
mMSC
mESC
mESC (Zhao)
ES
ESMS
MS
MSLS
LS
UND
0
1x10⁹
2x10⁹
3x10⁹
Genome covered
e.
MSO 6h
MSO 5h
S2
MSO 2h
MSO4h
G1
S1
MSO 12h
G2/M
MSO 10h
S4
MSO 7h
MSO 8h
S3
MSO 8h
MSO 7h
S4
MSO 10h
G2/M
MSO 12h
S1
G1
MSO 4h
MSO 2h
S2
MSO 5h
MSO 6h
−1.00
−0.75
−0.50
−0.25
0.00
0.25
0.50
0.75
1.00

Supplementary Figure 1. Kinetics of progression through S phase.

a, EdU pulse chase experiment monitoring progression through S phase by flow cytometry. Asynchronous cells were pulsed with EdU for 30 minutes, washed and released into fresh media. Cells were collected at the indicated time points. Progression through S phase could be monitored for both the EdU-positive cells and the cells that were not in S phase during the EdU pulse (EdU-negative cells; oblique arrows at the 8 h time point). For mESCs, vertical arrows at the 9 and 12 h time points indicate cells that entered S phase of a second cell cycle. EdU intensity is shown in the vertical y-axis and propidium iodide intensity (horizontal x-axis) was used as a marker of DNA content (ploidy). **b, c**, Genome-wide RT profile, as determined by Repli-seq. (b) Unsynchronized mESCs, MEFs and mMSCs were separated in fractions according to DNA content by flow sorting. (c) Repli-seq profiles of the indicated cell fractions. RT domains and annotated genes are indicated as in Fig. 1c. For mESCs, the RT domains determined from a published dataset (RT Zhao) [19] are also shown, revealing high similarity to the data obtained here. Orange open arrowheads show replication activity in late RT domains corresponding to putative IZs. A broader pattern of replication activity throughout the same RT domain (probably due to multiple stochastic firing) is marked with an orange open box in MEF and MSC. **d**, Genome-coverage by the RT domains in the different cell types and in [19]. ES, early S phase; ESMS, early to mid S transition; MS, mid S; MSLS, mid to late S transition; LS, late S; UND, undefined. Note that the analysis of IZs in this study did not include the ESMS, MSLS and UND regions, since these regions encompassed a small fraction of the genome and could not be unambiguously assigned to well-defined early, mid or late replicating domains. **e**, Clustering and correlation of genome-wide mESC EdU-seq and Repli-seq profiles. The data were obtained from the experiments outlined in Fig. 1b and Supplementary Fig. 1b.

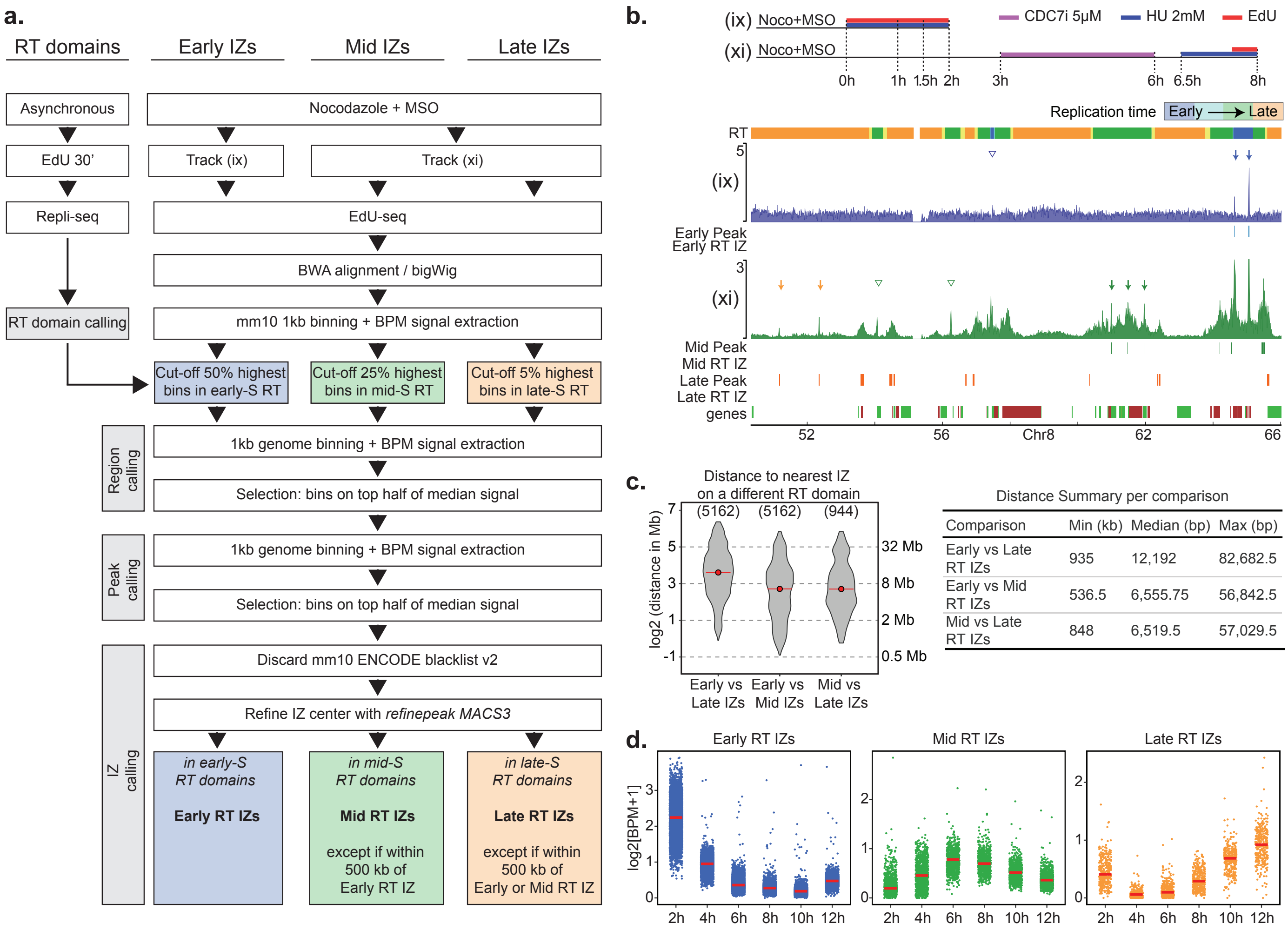

Supplementary Figure 2. IZ peak calling.

a, Workflow of IZ peak identification in mESC. **b**, Example of a region in chromosome 8 showing EdU-seq signal as in tracks (ix) and (xi) from Fig. 1e and f. Open arrowheads show putative mid RT IZs that were not considered by our stringent peak calling approach. The tracks below the EdU-seq show early, mid and late RT IZs before (preliminary) and after (definitive) refinement. The track above shows RT domains from the mESC Repli-seq experiments. **c**, Violin plots and a table showing the distribution of the distances of the closest pairwise comparisons of IZs belonging to different RT domain groups. In the plots, the numbers of computed early and mid IZs are shown in parentheses. In the table, the “min” and “max” columns indicate the closest and farthest distances between two IZs belonging to different RT domains. Source data are provided as a Source Data file. **d**, Individual EdU signal plotting of every identified early, mid and late RT IZ at every time point of the EdU-seq time-course. Source data are provided as a Source Data file.

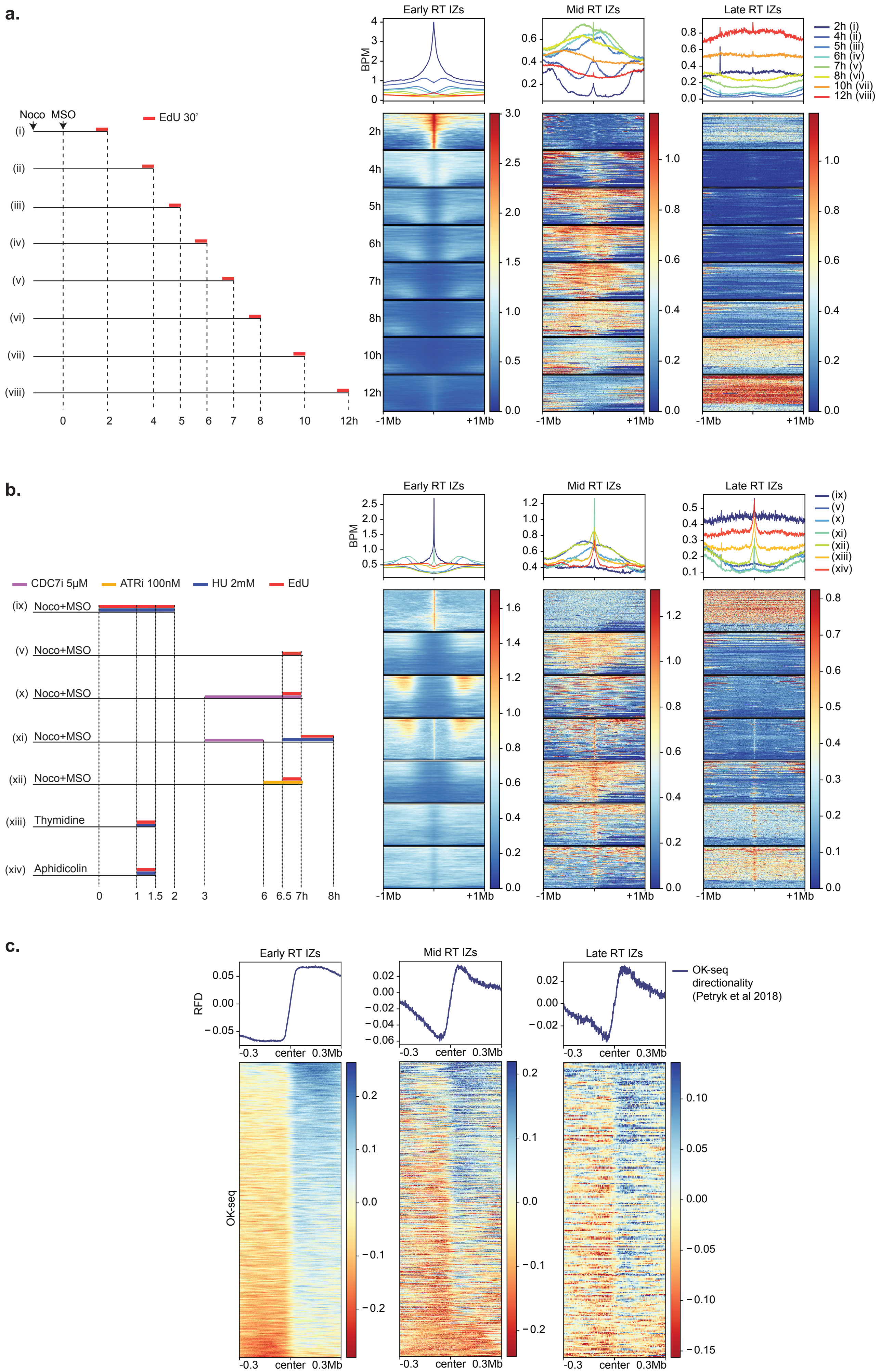
a.
Noco MSO
EdU 30'
(i)
(ii)
(iii)
(iv)
(v)
(vi)
(vii)
(viii)
0 2 4 5 6 7 8 10 12h
Early RT IZs
Mid RT IZs
Late RT IZs
BPM
2h (i)
4h (ii)
5h (iii)
6h (iv)
7h (v)
8h (vi)
10h (vii)
12h (viii)
2h 4h 5h 6h 7h 8h 10h 12h
-1Mb +1Mb
b.
CDC7i 5μM
ATRi 100nM
HU 2mM
EdU
(ix) Noco+MSO
(v) Noco+MSO
(x) Noco+MSO
(xi) Noco+MSO
(xii) Noco+MSO
(xiii) Thymidine
(xiv) Aphidicolin
0 1 1.5 2 3 6 6.5 7h 8h
Early RT IZs
Mid RT IZs
Late RT IZs
BPM
(ix)
(v)
(x)
(xi)
(xii)
(xiii)
(xiv)
-1Mb +1Mb
c.
Early RT IZs
Mid RT IZs
Late RT IZs
RFD
OK-seq
-0.3 center 0.3Mb
OK-seq
directionality
(Petryk et al 2018)

Supplementary Figure 3. Genome-wide analysis of bona-fide early, mid and late RT IZs.
a, Overlay of average EdU-seq signal (BPM) from the mESC time-course experiment shown in Fig. 1b, c around the identified mESC early, mid and late RT IZ. **b**, Overlay of average EdU-seq signal (BPM) from the experiments shown in Fig. 1e, f around the identified mESC early, mid and late RT IZ. Note that late RT IZs show clear isolated signal for samples (xi-xiv). **c**, Overlay of average replication fork directionality (RFD) calculated from OK-seq data [18] around identified early, mid and late RT IZs in mESC.

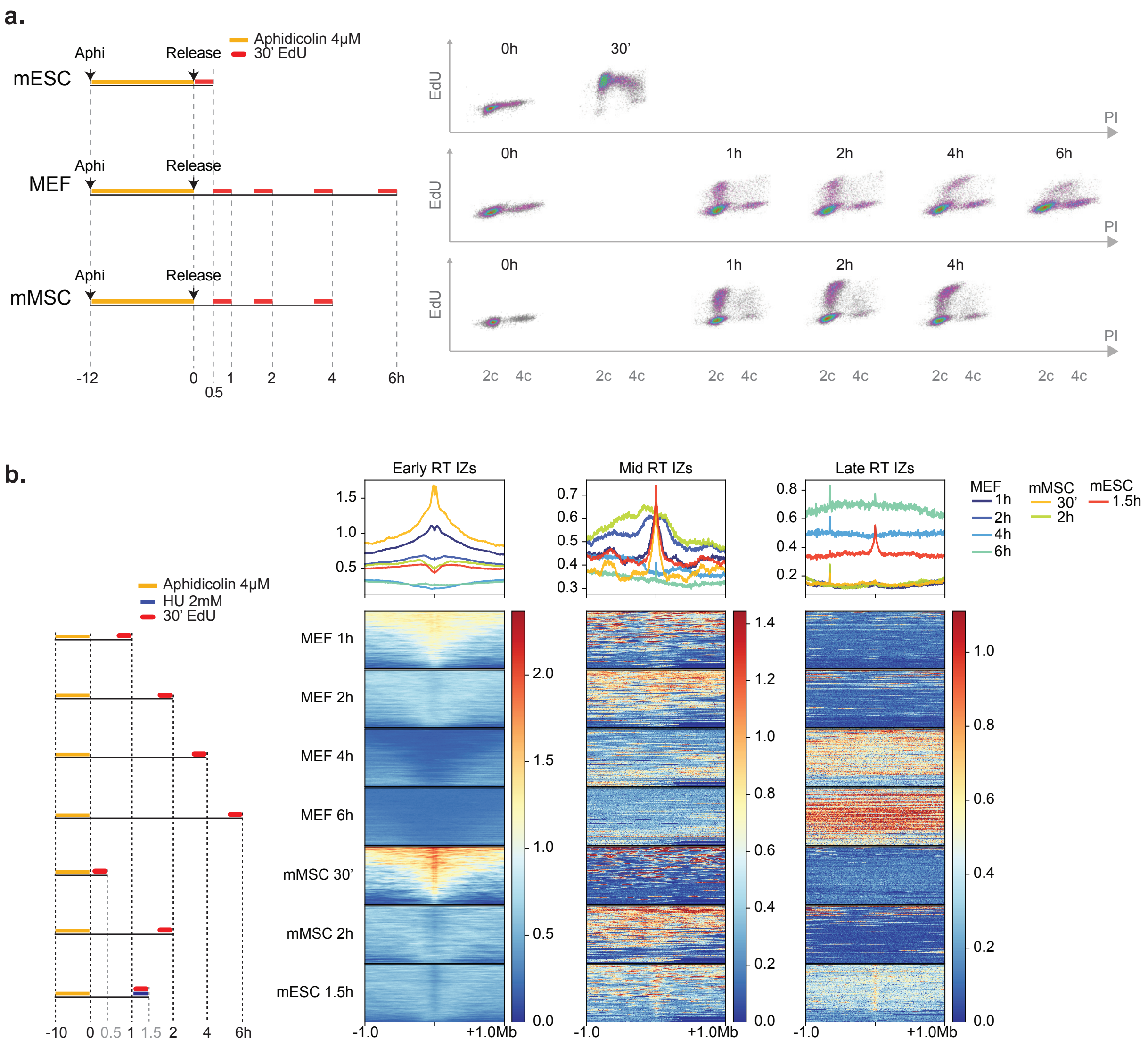

Supplementary Figure 4. Comparison of mESCs, MEFs and mMSCs.
a, FACS scatterplots showing similar dynamics in EdU (y-axis) and PI incorporation (x-axis) between mESC, MEF and mMSC when these cells have been released from an aphidicolin treatment. On the left, experimental scheme associated to the FACS experiment. **b**, Overlay of average EdU-seq signal (BPM) from the mESC, MEF and mMSC aphidicolin experiments centered around mESC bona-fide RT IZs.

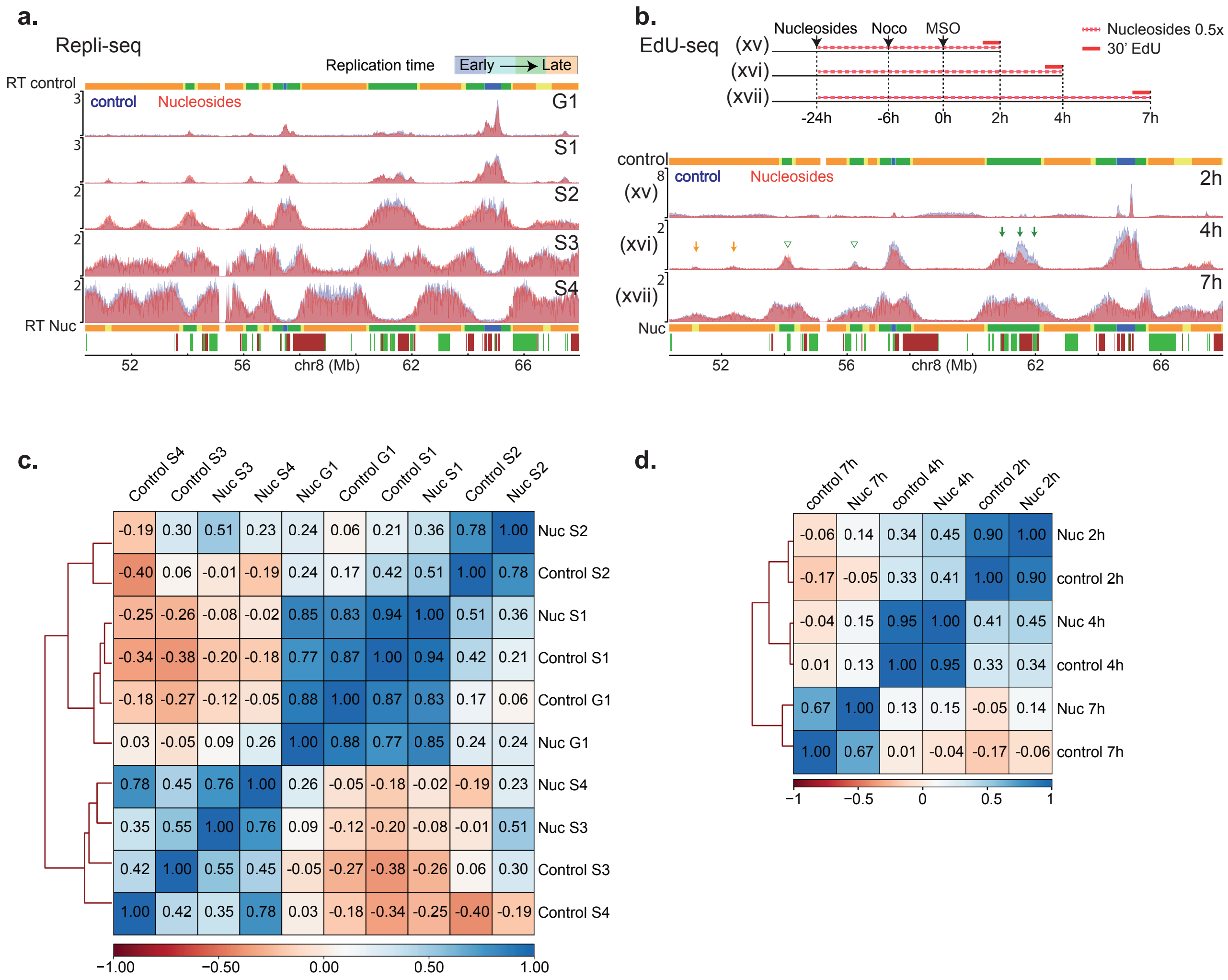

Supplementary Figure 5. Lack of effect of increased concentration of nucleosides on RT.
a, Overlay of mESC Repli-seq profiles exploring the effect of adding nucleosides (0.5x Embryomax) to the tissue culture media. RT domains and annotated genes are indicated as in Fig. 1c. **b**, Overlay of mESC EdU-seq profiles exploring the effect of adding nucleosides (0.5x Embryomax) to the tissue culture media. RT domains and annotated genes are indicated as in Fig. 1c. Top: experimental outline. **c**, Clustering and correlation of genome-wide mESC Repli-seq profiles exploring the effect of adding nucleosides (Nuc, 0.5x Embryomax) to the tissue culture media. The data are derived from the experiment shown in panel (a). **d**, Clustering and correlation of genome-wide mESC EdU-seq profiles exploring the effect of adding nucleosides (Nuc, 0.5x Embryomax) to the tissue culture media. The data are derived from the experiment shown in panel (b).

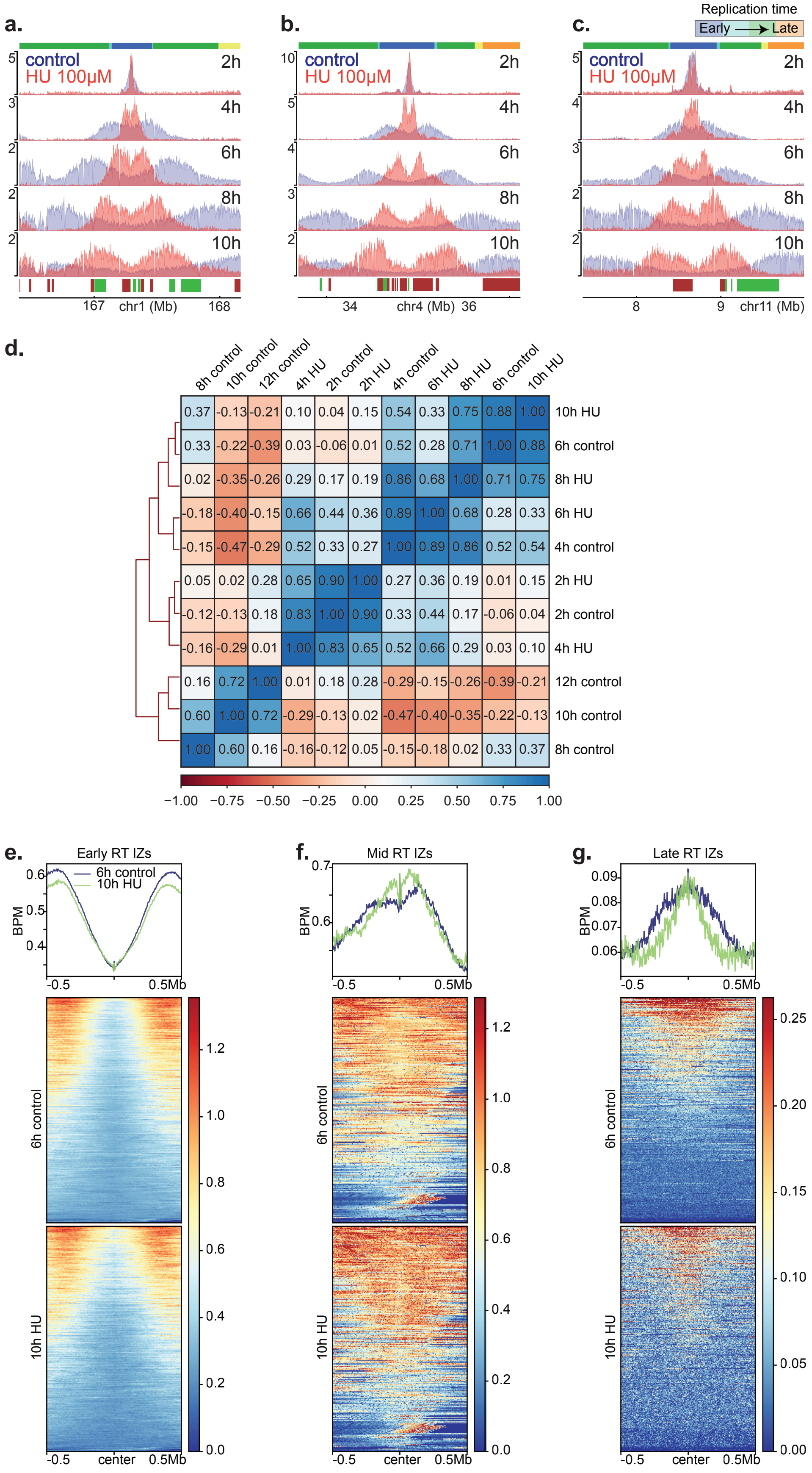

Supplementary Figure 6. Decreased nucleotide levels slow S phase progression without affecting relative RT. **a-c**, Overlay of mESC EdU-seq profiles exploring the effect of adding HU to the tissue culture media. Three different genomic regions are shown. RT domains and annotated genes are indicated as in Fig. 1c. **d**, Clustering and correlation of genome-wide mESC EdU-seq profiles exploring the effect of adding HU (100 µM) to the tissue culture media. The data are derived from the experiment shown in panels (a-c). **e-g**, Average EdU-seq signal around early, mid and late RT IZs from control and HU-treated mESCs harvested 6 and 10 h after MSO, respectively. The heatmaps below indicate the EdU-seq signal at each RT IZ. The data are derived from the experiment shown in panels (a-c).

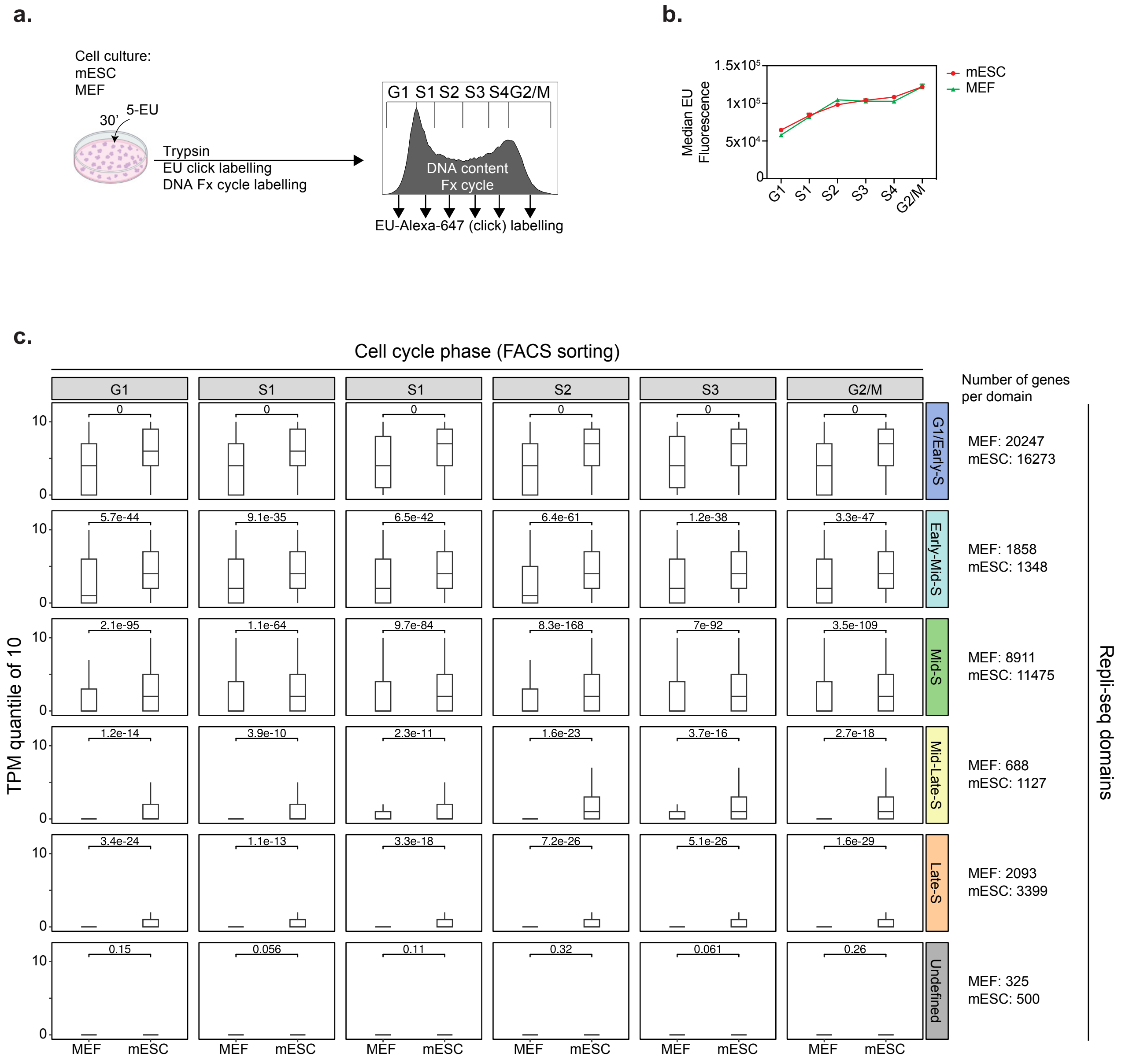

Supplementary Figure 7. Higher specific transcriptional output of mESC.
a, Experimental scheme of total nascent transcription measured by FACS for mESC and MEF (only the mESC PI distribution is shown). EU was added for 30' to asynchronous cell cultures, which were then sorted. EU was identified through a click reaction with an Alexa-647 biotin-conjugated antibody. **b**, Median EU Alexa 647 fluorescence of each sorted fraction for mESC and MEF. **c**, Distribution of nascent transcription in mESC and MEF for genes located in each RT domain (early-S, early-mid-S, mid-S, mid-late-S, late-S and undefined regions), along the cell cycle (sorted fractions from G1 to G2/M). On the right, the number of the genes considered in each RT domain is reported for each cell type. RNA signal is shown as TPM of quantile 10. Values represent the FDR corrected p-values of unpaired t-test of one experiment comparing mESC and MEF. Box plots show the median (centre line), the 25th (Q1) and 75th (Q3) percentiles (bounds of the box), and the minimum and maximum values within 1.5x the interquartile range (whiskers). Points outside this range are plotted individually as outliers. Source data are provided as a Source Data file.

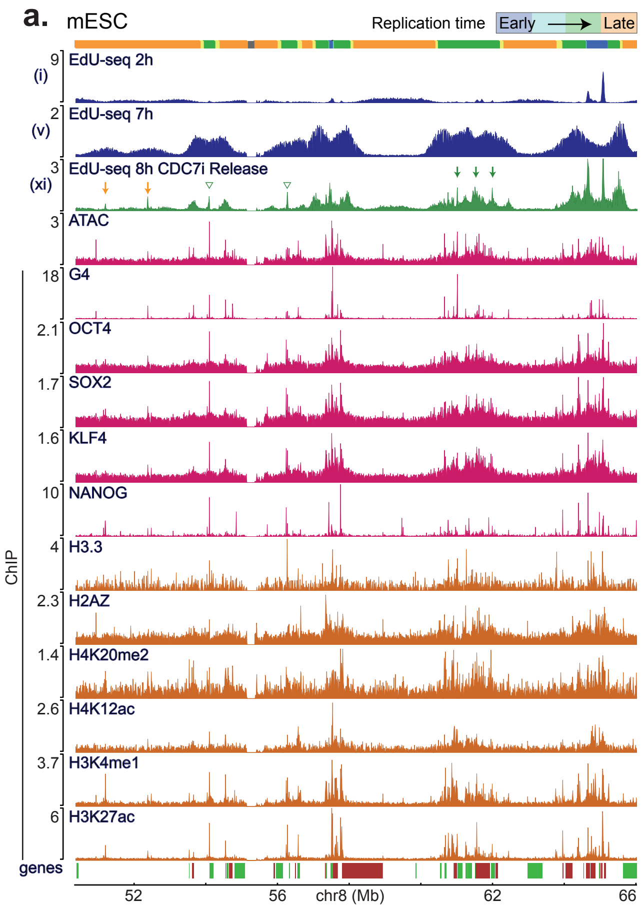

b.

Histone Mark	Group 1	Group 2	p-value
H2AZ	Control regions	Early RT IZs	1.32E-46
H2AZ	Control regions	Mid RT IZs	1.29E-90
H2AZ	Control regions	Late RT IZs	3.83E-16
H2AZ	Early RT IZs	Mid RT IZs	1.61E-12
H2AZ	Early RT IZs	Late RT IZs	8.63E-05
H2AZ	Mid RT IZs	Late RT IZs	1.47E-23
H3.3	Control regions	Early RT IZs	2.89E-178
H3.3	Control regions	Mid RT IZs	7.66E-90
H3.3	Control regions	Late RT IZs	2.70E-53
H3.3	Early RT IZs	Mid RT IZs	4.79E-36
H3.3	Early RT IZs	Late RT IZs	9.88E-29
H3.3	Mid RT IZs	Late RT IZs	0.00034771
H3K27ac	Control regions	Early RT IZs	2.25E-91
H3K27ac	Control regions	Mid RT IZs	2.81E-82
H3K27ac	Control regions	Late RT IZs	1.61E-63
H3K27ac	Early RT IZs	Mid RT IZs	3.25E-07
H3K27ac	Early RT IZs	Late RT IZs	3.59E-05
H3K27ac	Mid RT IZs	Late RT IZs	0.92002609
H3K4me1	Control regions	Early RT IZs	8.78E-128
H3K4me1	Control regions	Mid RT IZs	7.04E-88
H3K4me1	Control regions	Late RT IZs	2.50E-52
H3K4me1	Early RT IZs	Mid RT IZs	1.69E-06
H3K4me1	Early RT IZs	Late RT IZs	1.17E-08
H3K4me1	Mid RT IZs	Late RT IZs	0.02916766
H4K20me	Control regions	Early RT IZs	2.10E-184
H4K20me	Control regions	Mid RT IZs	9.78E-87
H4K20me	Control regions	Late RT IZs	6.93E-31
H4K20me	Early RT IZs	Mid RT IZs	8.73E-26
H4K20me	Early RT IZs	Late RT IZs	6.40E-27
H4K20me	Mid RT IZs	Late RT IZs	3.02E-06

Supplementary Figure 8. Open chromatin marks and recruitment of pluripotency factors at IZs.
a, EdU-seq profiles of mESCs (from Fig. 1e, f) highlighting mid and late RT IZs (green and orange arrows, respectively) aligned to pluripotency factor and histone mark ChIP-seq profiles over a representative genomic region. RT domains and gene annotation are indicated as in Fig. 1c. **b**, Statistical analysis of histone mark enrichment around IZs (+/- 300 kb). Wilcoxon rank-sum test.

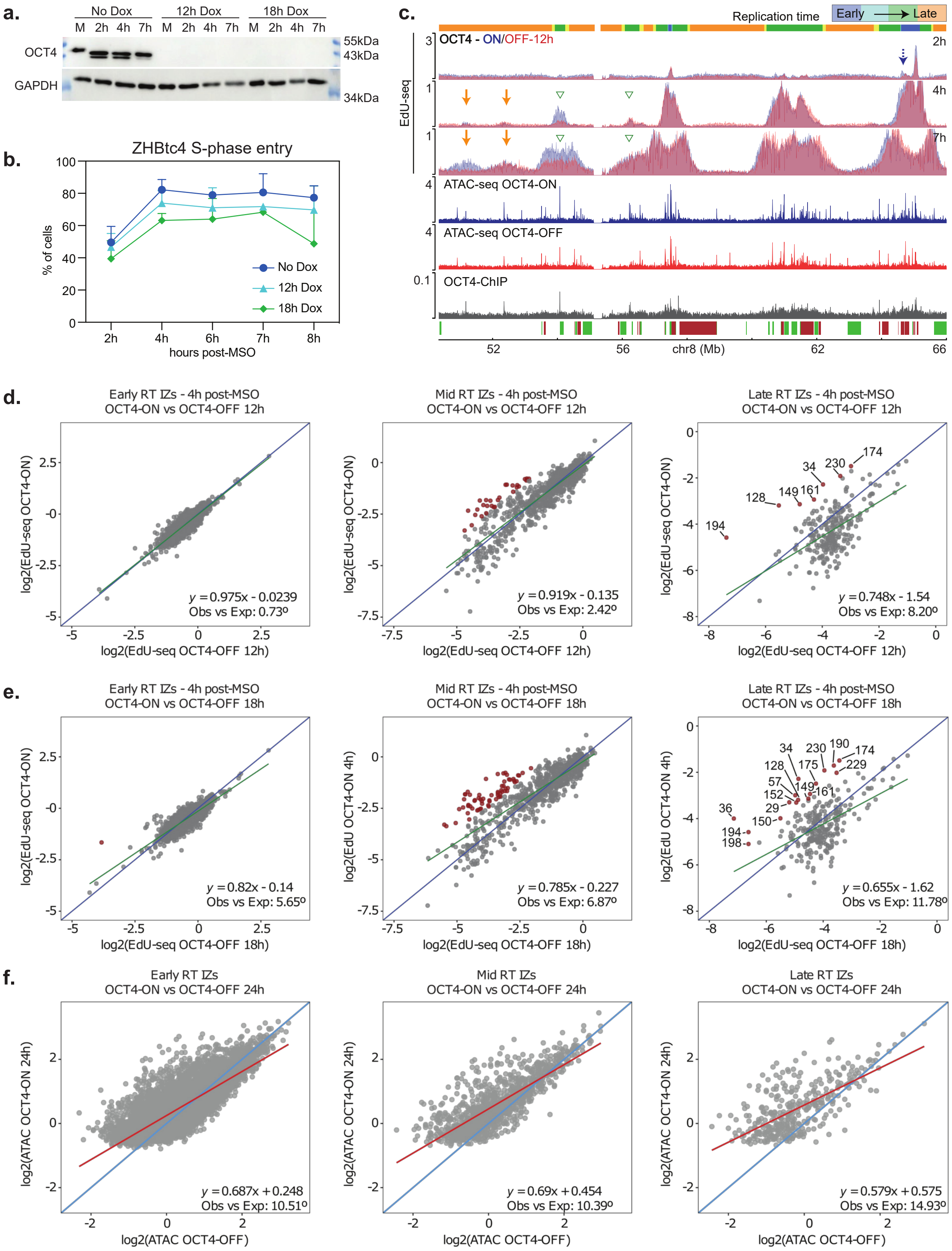
a.
No Dox
12h Dox
18h Dox
M 2h 4h 7h M 2h 4h 7h M 2h 4h 7h
OCT4
GAPDH
55kDa
43kDa
34kDa
b.
ZHBtc4 S-phase entry
% of cells
hours post-MSO
No Dox
12h Dox
18h Dox
c.
Replication time
Early
Late
OCT4 - ON/OFF-12h
EdU-seq
2h
4h
7h
ATAC-seq OCT4-ON
ATAC-seq OCT4-OFF
OCT4-ChIP
chr8 (Mb)
d.
Early RT IZs - 4h post-MSO
OCT4-ON vs OCT4-OFF 12h
y = 0.975x - 0.0239
Obs vs Exp: 0.73º
Mid RT IZs - 4h post-MSO
OCT4-ON vs OCT4-OFF 12h
y = 0.919x - 0.135
Obs vs Exp: 2.42º
Late RT IZs - 4h post-MSO
OCT4-ON vs OCT4-OFF 12h
y = 0.748x - 1.54
Obs vs Exp: 8.20º
log2(EdU-seq OCT4-ON)
log2(EdU-seq OCT4-OFF 12h)
e.
Early RT IZs - 4h post-MSO
OCT4-ON vs OCT4-OFF 18h
y = 0.82x - 0.14
Obs vs Exp: 5.65º
Mid RT IZs - 4h post-MSO
OCT4-ON vs OCT4-OFF 18h
y = 0.785x - 0.227
Obs vs Exp: 6.87º
Late RT IZs - 4h post-MSO
OCT4-ON vs OCT4-OFF 18h
y = 0.655x - 1.62
Obs vs Exp: 11.78º
log2(EdU-seq OCT4-ON)
log2(EdU OCT4-ON 4h)
log2(EdU-seq OCT4-OFF 18h)
f.
Early RT IZs
OCT4-ON vs OCT4-OFF 24h
y = 0.687x + 0.248
Obs vs Exp: 10.51º
Mid RT IZs
OCT4-ON vs OCT4-OFF 24h
y = 0.69x + 0.454
Obs vs Exp: 10.39º
Late RT IZs
OCT4-ON vs OCT4-OFF 24h
y = 0.579x + 0.575
Obs vs Exp: 14.93º
log2(ATAC OCT4-ON 24h)
log2(ATAC OCT4-OFF)

Supplementary Figure 9. OCT4 enhances firing of select mid and late RT IZs.

a, Immunoblot showing effective downregulation of OCT4 levels in ZHBTc4 cells treated with doxycycline (Dox). Cells were harvested at the time of MSO or 2, 4 and 7 h later. The experimental outline is shown in Fig. 5b. The presented blot is a representative image from two comparable experiments. Source data are provided as a Source Data file. **b**, Timing of entry into S phase, as monitored by flow cytometry analysis of the fraction of EdU-positive cells at the indicated time points after MSO. ZHBTc4 cells were treated as shown in the experimental outline of Fig. 5b. Source data are provided as a Source Data file. **c**, EdU-seq profiles of ZHBTc4 cells at the indicated times after MSO or after release from a thymidine block. Profiles with OCT4 expression turned ON or OFF (doxycycline added 12 h before MSO) are compared. Plots below the EdU-seq profiles: ATAC-seq profiles from asynchronous ZHBTc4 cells with OCT4 expression turned ON or OFF [34] and OCT4 ChIP-seq profile from asynchronous mESCs [69]. Putative mid and selected late RT IZs are indicated by green arrowheads and orange arrows, respectively. Blue dashed-arrow shows an affected early RT IZ. RT domains and gene annotation are indicated as in Fig. 1c. **d**, **e**, Scatterplots of EdU-seq signals 4h after MSO at early, mid and late RT IZs of ZHBTc4 cells expressing (ON) versus not expressing (OFF) OCT4. OCT4 expression was suppressed by administering doxycycline 12 h (e) and 18 h (f) before MSO. The EdU-seq signal for each IZ was computed as BPM in a region +/-5 kb around the centre of the IZ and transformed into log2. The diagonal blue lines indicate a hypothetical perfect correlation; the green lines show the trend of the real data; the angle between the lines was calculated to highlight the differences. All IZs with ON/OFF rates >2.5 are highlighted in red. Some late RT IZs are numbered for referencing. These results mimic those in Fig. 5e. **f**, Scatterplots of ATAC-seq signals at early, mid and late RT IZs, plotted as in (Supplementary Fig. 9e, f). At most late RT IZs, there is a decrease in the ATAC-seq signal, when OCT4 expression was turned off. The ATAC-seq data are from [34]. These results mimic those in Fig. 5f.
