# Supplementary material for: OCT4 enhances the firing efficiency of late DNA replication origins in mouse embryonic stem cells
Source: Nat Commun. 2026 Jan 15;17:1686. doi: 10.1038/s41467-026-68389-1 (PMC12910074; doi:10.1038/s41467-026-68389-1)
Supplement: Supplementary file 2 — Description of Additional Supplementary Files [file 41467_2026_68389_MOESM2_ESM.pdf]

Description of Additional Supplementary Files

Supplementary Data 1. It includes:

- List of Early RT IZs. List of IZs identified in early-S RT domains in mESC.
- List of Mid RT IZs. List of IZs identified in mid-S RT domains in mESC.
- List of Late RT IZs. List of IZs identified in late-S RT domains in mESC.
- Selected IZs fork progression. List of isolated early RT IZs that were used to calculate fork speed.
- mESC RT domains. RT domains identified in Repli-seq experiments performed in mESC.
- MEF RT domains. RT domains identified in Repli-seq experiments performed in MEF.
- mMSC RT domains. RT domains identified in Repli-seq experiments performed in mMSC.
- EdU-seq samples. List of samples (and replicates) of EdU-seq experiments done and used in the present study.
- Repli-seq samples. List of samples (and replicates) of EdU-seq experiments done and used in the present study.
- EU-seq samples. List of samples (and replicates) of EU-seq experiments done and used in the present study.
