# Supplementary material for: OCT4 enhances the firing efficiency of late DNA replication origins in mouse embryonic stem cells
Source: Nat Commun. 2026 Jan 15;17:1686. doi: 10.1038/s41467-026-68389-1 (PMC12910074; doi:10.1038/s41467-026-68389-1)
Supplement: Supplementary file 4 — Reporting Summary [file 41467_2026_68389_MOESM4_ESM.pdf]

nature portfolio

Corresponding author(s): Thanos Halazonetis

Last updated by author(s): 26/11/25

Reporting Summary

Nature Portfolio wishes to improve the reproducibility of the work that we publish. This form provides structure for consistency and transparency in reporting. For further information on Nature Portfolio policies, see our Editorial Policies and the Editorial Policy Checklist.

Statistics

For all statistical analyses, confirm that the following items are present in the figure legend, table legend, main text, or Methods section.

n/a	Confirmed	
[ ]	[x]	The exact sample size (*n*) for each experimental group/condition, given as a discrete number and unit of measurement
[ ]	[x]	A statement on whether measurements were taken from distinct samples or whether the same sample was measured repeatedly
[ ]	[x]	The statistical test(s) used AND whether they are one- or two-sided<br>*Only common tests should be described solely by name; describe more complex techniques in the Methods section.*
[x]	[ ]	A description of all covariates tested
[ ]	[x]	A description of any assumptions or corrections, such as tests of normality and adjustment for multiple comparisons
[ ]	[x]	A full description of the statistical parameters including central tendency (e.g. means) or other basic estimates (e.g. regression coefficient) AND variation (e.g. standard deviation) or associated estimates of uncertainty (e.g. confidence intervals)
[ ]	[x]	For null hypothesis testing, the test statistic (e.g. *F*, *t*, *r*) with confidence intervals, effect sizes, degrees of freedom and *P* value noted<br>*Give P values as exact values whenever suitable.*
[x]	[ ]	For Bayesian analysis, information on the choice of priors and Markov chain Monte Carlo settings
[x]	[ ]	For hierarchical and complex designs, identification of the appropriate level for tests and full reporting of outcomes
[ ]	[x]	Estimates of effect sizes (e.g. Cohen's *d*, Pearson's *r*), indicating how they were calculated

Our web collection on statistics for biologists contains articles on many of the points above.

Software and code

Policy information about availability of computer code

Data collection	Our own data was downloaded from the internal server (UHTS-LIMS) of the University of Geneva genomics platform.<br>Public data was downloaded from ENA browser.
Data analysis	FastQ files of the sequencing reads were aligned on the mouse reference genome NCBI Build GRCm38/mm10 using the Burrows-Wheeler Alignment tool v.0.7.17. The resulting SAM files were converted to BAM files keeping only the uniquely mapped reads. Samtools v1.9 was used to sort, remove the PCR duplicates and index the BAM file (https://arxiv.org/abs/1303.3997). The processed BAM files have been transformed to coverage bigWig tracks using the bamCoverage function of deeptools v.3.5.1. The coverage was calculated as the normalized number of reads per bins per million mapped reads (BPM) with minimum mapping quality of 30 (http://doi.org/10.1093/nar/gkw257).<br>For the characterization of the replication timing domains of mESCs, MEFs and MSC the processed BAM files of Repli-seq experiment were given as input to the Repliscan tool (Zynda et al., 2017) using a window size of 10kb.<br>The bash script for peak calling can be downloaded from github (https://github.com/VSDionellis/OCT4_ESC_IZ_replication).

For manuscripts utilizing custom algorithms or software that are central to the research but not yet described in published literature, software must be made available to editors and reviewers. We strongly encourage code deposition in a community repository (e.g. GitHub). See the Nature Portfolio guidelines for submitting code & software for further information.

Data

Policy information about availability of data

All manuscripts must include a data availability statement. This statement should provide the following information, where applicable:

- Accession codes, unique identifiers, or web links for publicly available datasets
- A description of any restrictions on data availability
- For clinical datasets or third party data, please ensure that the statement adheres to our policy

All the data produced in this study has been deposited in GEO database under accession numbers:
GSE271841 (https://www.ncbi.nlm.nih.gov/geo/query/acc.cgi?acc=GSE271841)
GSE271846 (https://www.ncbi.nlm.nih.gov/geo/query/acc.cgi?acc=GSE271846)
GSE271847 (https://www.ncbi.nlm.nih.gov/geo/query/acc.cgi?acc=GSE271847)

Research involving human participants, their data, or biological material

Policy information about studies with human participants or human data. See also policy information about sex, gender (identity/presentation), and sexual orientation and race, ethnicity and racism.

Reporting on sex and gender	No human material was used in this study.
Reporting on race, ethnicity, or other socially relevant groupings	No human material was used in this study.
Population characteristics	No human material was used in this study.
Recruitment	No human material was used in this study.
Ethics oversight	No human material was used in this study.

Note that full information on the approval of the study protocol must also be provided in the manuscript.

Field-specific reporting

Please select the one below that is the best fit for your research. If you are not sure, read the appropriate sections before making your selection.

[x] Life sciences [ ] Behavioural & social sciences [ ] Ecological, evolutionary & environmental sciences

For a reference copy of the document with all sections, see nature.com/documents/nr-reporting-summary-flat.pdf

Life sciences study design

All studies must disclose on these points even when the disclosure is negative.

Sample size	No statistical method was used to predetermine sample size. The reasons not to perform more than one replicate in some conditions were based in the fact that their outcomes could be directly cross-checked with similar conditions rendering comparable results.
Data exclusions	No data were excluded from the analyses.
Replication	All replicates yielded comparable results wiith non-abherant standard deviations
Randomization	Cells were grown in independent plates, on different days. Sample processing took place in tubes marked with arbitrary numbers that did not reference the treatment. Samples were sequenced along different months.
Blinding	Blinding was not relevant for this study.

Reporting for specific materials, systems and methods

We require information from authors about some types of materials, experimental systems and methods used in many studies. Here, indicate whether each material, system or method listed is relevant to your study. If you are not sure if a list item applies to your research, read the appropriate section before selecting a response.

Materials & experimental systems

n/a	Involved in the study
☐	☒ Antibodies
☐	☒ Eukaryotic cell lines
☒	☐ Palaeontology and archaeology
☒	☐ Animals and other organisms
☒	☐ Clinical data
☒	☐ Dual use research of concern
☒	☐ Plants

Methods

n/a	Involved in the study
☐	☒ ChIP-seq
☐	☒ Flow cytometry
☒	☐ MRI-based neuroimaging

Antibodies

Antibodies used

gH2AX (1:1000, Upstate, cat. no. 05-636);
p-CHK1 (1:1000, Cell signalling cat. no. 2341);
PCNA (1:1000, Millipore cat. no. MABE288);
OCT3/4 (1:500, Santa Cruz, cat. no. sc5279);
GAPDH (1:1000, Abcam, cat. no. ab181602)

Validation

gH2AX; information from Merck's website:
Immunoblot Analysis : 0.05-1 µg/ml of this antibody detected phosphorylated histone H2A.X (Ser139) in acid extracted histone lysates from Jurkat cells treated with 0.5 µM staurosporine (Catalog # 19-123). Example of publications: Hatano et al 2015.NAR. 43: 4075-86.

p-CHK1: A polyclonal rabbit antibody, supplied by Cell Signaling Technology, raised against Serine/threonine-protein kinase Chk1 (Human), cited in 402 publications, with 65 published images. Applications used include WB, ICC-IF, IF, FC/FACS, and 5 others.

PCNA: A monoclonal mouse antibody, supplied by Millipore-Sigma, raised against Proliferating cell nuclear antigen (Human), cited in 9 publications. Applications used include WB, IHC, and IHC-IF.

OCT3/4; information from Santa Cruz's website:
Oct3/4 Antibody (C-10) is a mouse monoclonal IgG2b κ Oct3/4 antibody, cited in 2,667 publications. Example: Niwa, H., et al. 2000. Nat Genet. 24: 372-6. PMID: 10742100

GAPDH; information from Abcam's website:
Rabbit Recombinant Monoclonal GAPDH antibody. Suitable for IP, WB, ICC/IF, Flow Cyt (Intra), IHC-P and reacts with Human, Mouse, Rat, African green monkey, Zebrafish, Xenopus tropicalis, Chicken samples. Cited in 2473 publications. Example: Hao, X-X, et al. 2016. Mol Reprod Dev. 83(7):615-23. PMID: 27265621

Eukaryotic cell lines

Policy information about cell lines and Sex and Gender in Research

Cell line source(s)

mESC cell line (Merck Millipore). CMTI-1. Embryonic Stem Cell Lines derived from murine 129/SVEV. Obtained from male mouse embryos.
MEF primary cell culture. Mouse Embryonic Fibroblasts. Cells extracted from sexually unclassified E13-14 mouse embryos (C57/Bl6).
mBM-MSC. Mouse bone-marrow mesenchymal stem cells. Obtained from laboratory Prof Ventura Laboratory (University of Barcelona). Female mouse.
ZHBTc4. Mouse embryonic stem cell mouse. Obtained from male mouse embryos. Niwa H et al. 2000. Nat Genet 24(4):372-6. PMID: 10742100.

Authentication

CMTI-1. Authenticated by vendor.
MEF. not-authenticated.
mBM-MSC. Not authenticated.
ZHBTc4. Not authenticated.

Mycoplasma contamination

CMTI-1. Mycoplasma negative (MyoGenie Rapid MORV0011-50).
MEF. Mycoplasma negative (MyoGenie Rapid MORV0011-50).
mBM-MSC. Mycoplasma positive (MyoGenie Rapid MORV0011-50).
ZHBTc4. Mycoplasma negative (MyoGenie Rapid MORV0011-50).

Commonly misidentified lines
(See ICLAC register)

No commonly misidentified cells were used in this study.

Plants

Seed stocks: No plants were used in this study.

Novel plant genotypes: No plants were used in this study.

Authentication: No plants were used in this study.

ChIP-seq

Data deposition

☒ Confirm that both raw and final processed data have been deposited in a public database such as GEO.

☒ Confirm that you have deposited or provided access to graph files (e.g. BED files) for the called peaks.

Data access links
May remain private before publication.

To review the datasets:
https://www.ncbi.nlm.nih.gov/geo/query/acc.cgi?acc=GSE271841
token: atovsaimdtiplkd
https://www.ncbi.nlm.nih.gov/geo/query/acc.cgi?acc=GSE271846
token: idehceeajbepnsn
https://www.ncbi.nlm.nih.gov/geo/query/acc.cgi?acc=GSE271847
token: efsrogecrfuxrwv

Files in database submission

Repliseq_mESC_G1
Repliseq_mESC_S1
Repliseq_mESC_S2
Repliseq_mESC_S3
Repliseq_mESC_S4
Repliseq_mESC_Nuc_G1
Repliseq_mESC_Nuc_S1
Repliseq_mESC_Nuc_S2
Repliseq_mESC_Nuc_S3
Repliseq_mESC_Nuc_S4
Repliseq_MEF_G1
Repliseq_MEF_S1
Repliseq_MEF_S2
Repliseq_MEF_S3
Repliseq_MEF_S4
Repliseq_mBMMSC_G1
Repliseq_mBMMSC_S1
Repliseq_mBMMSC_S2
Repliseq_mBMMSC_S3
Repliseq_mBMMSC_S4
EdUseq_mESC_MSO_2hEdUHU
EdUseq_mESC_MSO_2h_rep1
EdUseq_mESC_MSO_2h_rep2
EdUseq_mESC_MSO_4h_rep1
EdUseq_mESC_MSO_4h_rep2
EdUseq_mESC_MSO_5h_rep1
EdUseq_mESC_MSO_5h_rep2
EdUseq_mESC_MSO_6h_rep1a
EdUseq_mESC_MSO_6h_rep1bc
EdUseq_mESC_MSO_6h_rep2
EdUseq_mESC_MSO_6h_rep3
EdUseq_mESC_MSO_7h_rep1
EdUseq_mESC_MSO_7h_rep2
EdUseq_mESC_MSO_7h_rep3
EdUseq_mESC_MSO_7h_rep4
EdUseq_mESC_MSO_8h_rep1
EdUseq_mESC_MSO_8h_rep2

EdUseq_mESC_MSO_10h_rep1
EdUseq_mESC_MSO_10h_rep2
EdUseq_mESC_MSO_10h_rep3
EdUseq_mESC_MSO_12h_rep1
EdUseq_mESC_MSO_12h_rep2
EdUseq_mESC_Thy_60mR_30mEdUHU_rep1
EdUseq_mESC_Thy_60mR_30mEdUHU_rep2
EdUseq_mESC_Aphi_60mR_30mEdUHU_rep1
EdUseq_mESC_Aphi_60mR_30mEdUHU_rep2
EdUseq_mESC_MSO_CDC7i+HU_8h
EdUseq_mESC_MSO_lowHU_2h
EdUseq_mESC_MSO_lowHU_4h_rep1
EdUseq_mESC_MSO_lowHU_4h_rep2
EdUseq_mESC_MSO_lowHU_6h_rep1
EdUseq_mESC_MSO_lowHU_6h_rep2
EdUseq_mESC_MSO_lowHU_8h_rep1
EdUseq_mESC_MSO_lowHU_8h_rep2
EdUseq_mESC_MSO_lowHU_10h
EdUseq_mESC_MSO_Ai_2h
EdUseq_mESC_MSO_Ai_4h
EdUseq_mESC_MSO_Ai_6h
EdUseq_mESC_MSO_Ai_8h
EdUseq_mESC_MSO_Ai_10h
EdUseq_mESC_MSO_lowHU+Ai_4h
EdUseq_mESC_MSO_lowHU+Ai_6h
EdUseq_mESC_MSO_lowHU+Ai_8h
EdUseq_mESC_MSO_1hATRi_7h_rep1
EdUseq_mESC_MSO_1hATRi_7h_rep2
EdUseq_mESC_MSO_TAK_4h_merged
EdUseq_mESC_MSO_TAK_7h_merged
EdUseq_mESC_CDC7i_7h
EdUseq_mESC_MSO_12Thy_60mR_30mEdUHU
EdUseq_mESC_MSO_10TAK_60mR_30mEdUHU
EdUseq_mESC_MSO_Nuc_2h
EdUseq_mESC_MSO_Nuc_4h
EdUseq_mESC_MSO_Nuc_7h
EdUseq_mESC_MSO_TAK+RO_7h_merged
EdUseq_mESC_MSO_TAK+ATRi_7h_merged
EdUseq_mESC_MSO_TAK+ATRi+RO_7h_merged
EdUseq_ZHBTc4_noDox_MSO_2h_rep1
EdUseq_ZHBTc4_noDox_MSO_2h_rep2
EdUseq_ZHBTc4_noDox_MSO_4h_rep1
EdUseq_ZHBTc4_noDox_MSO_4h_rep2
EdUseq_ZHBTc4_noDox_MSO_7h_rep1
EdUseq_ZHBTc4_noDox_MSO_7h_rep2
EdUseq_ZHBTc4_noDox_MSO_7h_rep3
EdUseq_ZHBTc4_12Dox_MSO_2h_rep1
EdUseq_ZHBTc4_12Dox_MSO_2h_rep2
EdUseq_ZHBTc4_12Dox_MSO_4h_rep1
EdUseq_ZHBTc4_12Dox_MSO_4h_rep2
EdUseq_ZHBTc4_12Dox_MSO_4h_rep3
EdUseq_ZHBTc4_12Dox_MSO_7h_rep1
EdUseq_ZHBTc4_12Dox_MSO_7h_rep2
EdUseq_ZHBTc4_18Dox_MSO_2h_rep1
EdUseq_ZHBTc4_18Dox_MSO_2h_rep2
EdUseq_ZHBTc4_18Dox_MSO_2h_rep3
EdUseq_ZHBTc4_18Dox_MSO_4h_rep1
EdUseq_ZHBTc4_18Dox_MSO_4h_rep2
EdUseq_ZHBTc4_18Dox_MSO_4h_rep3
EdUseq_ZHBTc4_18Dox_MSO_7h_rep1
EdUseq_ZHBTc4_18Dox_MSO_7h_rep2
EdUseq_ZHBTc4_noDox_Thy_30R30E
EdUseq_ZHBTc4_24Dox_Thy_30R30E
EdUseq_MEF_Aphi_1h
EdUseq_MEF_Aphi_2h
EdUseq_MEF_Aphi_4h
EdUseq_MEF_Aphi_6h
EdUseq_mBMMSC_Aphi_30m
EdUseq_mBMMSC_Aphi_2h

EUseq_mESC_G1_rep1
EUseq_mESC_G1_rep2
EUseq_mESC_S1_rep1
EUseq_mESC_S1_rep2
EUseq_mESC_S2_rep1
EUseq_mESC_S2_rep2
EUseq_mESC_S3_rep1
EUseq_mESC_S3_rep2
EUseq_mESC_S4_rep1
EUseq_mESC_S4_rep2
Euseq_mESC_G2_M_rep1
Euseq_mESC_G2_M_rep2
EUseq_MEF_G1
EUseq_MEF_S1
EUseq_MEF_S2
EUseq_MEF_S3
EUseq_MEF_S4
Euseq_MEF_G2_M

Genome browser session
(e.g. UCSC)

Provide a link to an anonymized genome browser session for "Initial submission" and "Revised version" documents only, to enable peer review. Write "no longer applicable" for "Final submission" documents.

Methodology

Replicates

Repliseq_mESC_G1: 1 replicate
Repliseq_mESC_S1: 1 replicate
Repliseq_mESC_S2: 1 replicate
Repliseq_mESC_S3: 1 replicate
Repliseq_mESC_S4: 1 replicate
Repliseq_mESC_Nuc_G1: 1 replicate
Repliseq_mESC_Nuc_S1: 1 replicate
Repliseq_mESC_Nuc_S2: 1 replicate
Repliseq_mESC_Nuc_S3: 1 replicate
Repliseq_mESC_Nuc_S4: 1 replicate
Repliseq_MEF_G1: 1 replicate
Repliseq_MEF_S1: 1 replicate
Repliseq_MEF_S2: 1 replicate
Repliseq_MEF_S3: 1 replicate
Repliseq_MEF_S4: 1 replicate
Repliseq_mBMMSC_G1: 1 replicate
Repliseq_mBMMSC_S1: 1 replicate
Repliseq_mBMMSC_S2: 1 replicate
Repliseq_mBMMSC_S3: 1 replicate
Repliseq_mBMMSC_S4: 1 replicate
EdUseq_mESC_MSO_2h: 2 replicates
EdUseq_mESC_MSO_4h: 2 replicates
EdUseq_mESC_MSO_5h: 2 replicates
EdUseq_mESC_MSO_6h: 3 replicates
EdUseq_mESC_MSO_7h: 4 replicates
EdUseq_mESC_MSO_8h: 2 replicates
EdUseq_mESC_MSO_10h: 3 replicates
EdUseq_mESC_MSO_12h: 2 replicates
EdUseq_mESC_Thy_60mR_30mEdUHU: 2 replicates
EdUseq_mESC_Aphi_60mR_30mEdUHU: 2 replicates
EdUseq_mESC_MSO_CDC7i+HU_8h: 1 replicate
EdUseq_mESC_MSO_lowHU_2h: 1 replicate
EdUseq_mESC_MSO_lowHU_4h: 2 replicates
EdUseq_mESC_MSO_lowHU_6h: 2 replicates
EdUseq_mESC_MSO_lowHU_8h: 2 replicates
EdUseq_mESC_MSO_lowHU_10h: 1 replicate
EdUseq_mESC_MSO_Ai_2h: 1 replicate
EdUseq_mESC_MSO_Ai_4h: 1 replicate
EdUseq_mESC_MSO_Ai_6h: 1 replicate
EdUseq_mESC_MSO_Ai_8h: 1 replicate
EdUseq_mESC_MSO_Ai_10h: 1 replicate
EdUseq_mESC_MSO_lowHU+Ai_4h: 1 replicate
EdUseq_mESC_MSO_lowHU+Ai_6h: 1 replicate
EdUseq_mESC_MSO_lowHU+Ai_8h: 1 replicate
EdUseq_mESC_MSO_1hATRi_7h: 2 replicates

EdUseq_mESC_MSO_TAK_4h_merged: 1 replicate
EdUseq_mESC_MSO_TAK_7h_merged: 1 replicate
EdUseq_mESC_CDC7i_7h: 1 replicate
EdUseq_mESC_MSO_12Thy_60mR_30mEdUHU: 1 replicate
EdUseq_mESC_MSO_10TAK_60mR_30mEdUHU: 1 replicate
EdUseq_mESC_MSO_Nuc_2h: 1 replicate
EdUseq_mESC_MSO_Nuc_4h: 1 replicate
EdUseq_mESC_MSO_Nuc_7h: 1 replicate
EdUseq_mESC_MSO_TAK+RO_7h_merged: 1 replicate
EdUseq_mESC_MSO_TAK+ATRi_7h_merged: 1 replicate
EdUseq_mESC_MSO_TAK+ATRi+RO_7h_merged: 1 replicate
EdUseq_ZHBTc4_noDox_MSO_2h: 2 replicates
EdUseq_ZHBTc4_noDox_MSO_4h: 2 replicates
EdUseq_ZHBTc4_noDox_MSO_7h: 3 replicates
EdUseq_ZHBTc4_12Dox_MSO_2h: 2 replicates
EdUseq_ZHBTc4_12Dox_MSO_4h: 3 replicates
EdUseq_ZHBTc4_12Dox_MSO_7h: 2 replicates
EdUseq_ZHBTc4_18Dox_MSO_2h: 3 replicates
EdUseq_ZHBTc4_18Dox_MSO_4h: 3 replicates
EdUseq_ZHBTc4_18Dox_MSO_7h: 2 replicates
EdUseq_ZHBTc4_noDox_Thy_30R30E: 1 replicate
EdUseq_ZHBTc4_24Dox_Thy_30R30E: 1 replicate
EdUseq_MEF_Aphi_1h: 1 replicate
EdUseq_MEF_Aphi_2h: 1 replicate
EdUseq_MEF_Aphi_4h: 1 replicate
EdUseq_MEF_Aphi_6h: 1 replicate
EdUseq_mBMMSC_Aphi_30m: 1 replicate
EdUseq_mBMMSC_Aphi_2h: 1 replicate
EUseq_mESC_G1: 2 replicates
EUseq_mESC_S1: 2 replicates
EUseq_mESC_S2: 2 replicates
EUseq_mESC_S3: 2 replicates
EUseq_mESC_S4: 2 replicates
Euseq_mESC_G2_M: 2 replicates
EUseq_MEF_G1: 1 replicate
EUseq_MEF_S1: 1 replicate
EUseq_MEF_S2: 1 replicate
EUseq_MEF_S3: 1 replicate
EUseq_MEF_S4: 1 replicate

Sequencing depth

Sample Name ; Total reads ; Deduplicated and uniquely mapped
EdUseq_mBMMSC_Aphi_2h ; 11378230 ; 9423536
EdUseq_mBMMSC_Aphi_30m ; 15341621 ; 12238620
EdUseq_MEF_Aphi_1h ; 24546366 ; 19663887
EdUseq_MEF_Aphi_2h ; 24054929 ; 19543515
EdUseq_MEF_Aphi_4h ; 24543811 ; 14347190
EdUseq_MEF_Aphi_6h ; 17530312 ; 9804003
EdUseq_mESC_Aphi_60mR_30mEdUHU_rep1 ; 14654081 ; 8511783
EdUseq_mESC_Aphi_60mR_30mEdUHU_rep2 ; 24858364 ; 16002876
EdUseq_mESC_CDC7i_7h ; 35154823 ; 22465569
EdUseq_mESC_MSO_10h_rep1 ; 14327758 ; 9009411
EdUseq_mESC_MSO_10h_rep2 ; 22842747 ; 9558868
EdUseq_mESC_MSO_10h_rep3 ; 55265851 ; 22312744
EdUseq_mESC_MSO_10TAK_60mR_30mEdUHU ; 22198022 ; 15815558
EdUseq_mESC_MSO_12h_rep1 ; 17978258 ; 11872847
EdUseq_mESC_MSO_12h_rep2 ; 19035675 ; 7995142
EdUseq_mESC_MSO_12Thy_60mR_30mEdUHU ; 21763120 ; 13151983
EdUseq_mESC_MSO_1hATRi_7h_rep1 ; 12624826 ; 6816528
EdUseq_mESC_MSO_1hATRi_7h_rep2 ; 30124213 ; 12684226
EdUseq_mESC_MSO_2hEdUHU ; 12314638 ; 5113283
EdUseq_mESC_MSO_2h_rep1 ; 16341822 ; 11314458
EdUseq_mESC_MSO_2h_rep2 ; 17751043 ; 9439718
EdUseq_mESC_MSO_4h_rep1 ; 12826357 ; 10793349
EdUseq_mESC_MSO_4h_rep2 ; 9724948 ; 7737759
EdUseq_mESC_MSO_5h_rep1 ; 16994834 ; 11145316
EdUseq_mESC_MSO_5h_rep2 ; 9085651 ; 6705757
EdUseq_mESC_MSO_6h_rep1a ; 15389097 ; 9772960
EdUseq_mESC_MSO_6h_rep1bc ; 23273270 ; 9550357

EdUseq_mESC_MSO_6h_rep2 ; 11838772 ; 8367196
EdUseq_mESC_MSO_6h_rep3 ; 39119240 ; 17333639
EdUseq_mESC_MSO_7h_rep1 ; 20643098 ; 13802588
EdUseq_mESC_MSO_7h_rep2 ; 10458740 ; 6003508
EdUseq_mESC_MSO_7h_rep3 ; 21415810 ; 13367160
EdUseq_mESC_MSO_7h_rep4 ; 8533857 ; 5792188
EdUseq_mESC_MSO_8h_rep1 ; 16608416 ; 11114934
EdUseq_mESC_MSO_8h_rep2 ; 21600689 ; 9492791
EdUseq_mESC_MSO_Ai_10h ; 44989960 ; 18347647
EdUseq_mESC_MSO_Ai_2h ; 34758076 ; 16783293
EdUseq_mESC_MSO_Ai_4h ; 33324993 ; 17661141
EdUseq_mESC_MSO_Ai_6h ; 51633685 ; 21764946
EdUseq_mESC_MSO_Ai_8h ; 34872629 ; 14890417
EdUseq_mESC_MSO_CDC7i+HU_8h ; 50648407 ; 28776237
EdUseq_mESC_MSO_lowHU_10h ; 23561095 ; 11260125
EdUseq_mESC_MSO_lowHU_2h ; 19725087 ; 9023617
EdUseq_mESC_MSO_lowHU_4h_rep1 ; 14104106 ; 9061650
EdUseq_mESC_MSO_lowHU_4h_rep2 ; 26882493 ; 16332958
EdUseq_mESC_MSO_lowHU_6h_rep1 ; 15814660 ; 9725548
EdUseq_mESC_MSO_lowHU_6h_rep2 ; 26543513 ; 18010824
EdUseq_mESC_MSO_lowHU_8h_rep1 ; 19019757 ; 10366981
EdUseq_mESC_MSO_lowHU_8h_rep2 ; 32370890 ; 21851240
EdUseq_mESC_MSO_lowHU+Ai_4h ; 26626684 ; 15896305
EdUseq_mESC_MSO_lowHU+Ai_6h ; 29455017 ; 18549196
EdUseq_mESC_MSO_lowHU+Ai_8h ; 21875118 ; 13816044
EdUseq_mESC_MSO_Nuc_2h ; 52895811 ; 34633574
EdUseq_mESC_MSO_Nuc_4h ; 39784670 ; 29134292
EdUseq_mESC_MSO_Nuc_7h ; 43337865 ; 26190166
EdUseq_mESC_MSO_TAK_4h_merged ; 33267666 ; 26003725
EdUseq_mESC_MSO_TAK_7h_merged ; 97894713 ; 47998896
EdUseq_mESC_MSO_TAK+ATRi_7h_merged ; 68984331 ; 33322582
EdUseq_mESC_MSO_TAK+ATRi+RO_7h_merged ; 46550256 ; 23123469
EdUseq_mESC_MSO_TAK+RO_7h_merged ; 41662348 ; 26698581
EdUseq_mESC_Thy_60mR_30mEdUHU_rep1 ; 14369074 ; 8996059
EdUseq_mESC_Thy_60mR_30mEdUHU_rep2 ; 19018981 ; 11852081
EdUseq_ZHBTc4_12Dox_MSO_2h_rep1 ; 17843575 ; 12836923
EdUseq_ZHBTc4_12Dox_MSO_2h_rep2 ; 19369981 ; 12109134
EdUseq_ZHBTc4_12Dox_MSO_4h_rep1 ; 24845567 ; 19382582
EdUseq_ZHBTc4_12Dox_MSO_4h_rep2 ; 13748011 ; 9929821
EdUseq_ZHBTc4_12Dox_MSO_4h_rep3 ; 23054683 ; 13356856
EdUseq_ZHBTc4_12Dox_MSO_7h_rep1 ; 21415606 ; 13181934
EdUseq_ZHBTc4_12Dox_MSO_7h_rep2 ; 19439800 ; 9039594
EdUseq_ZHBTc4_18Dox_MSO_2h_rep1 ; 14025844 ; 10504496
EdUseq_ZHBTc4_18Dox_MSO_2h_rep2 ; 16041943 ; 12616884
EdUseq_ZHBTc4_18Dox_MSO_2h_rep3 ; 24015517 ; 14534299
EdUseq_ZHBTc4_18Dox_MSO_4h_rep1 ; 11451423 ; 8257717
EdUseq_ZHBTc4_18Dox_MSO_4h_rep2 ; 46533309 ; 29889755
EdUseq_ZHBTc4_18Dox_MSO_4h_rep3 ; 21460909 ; 12447108
EdUseq_ZHBTc4_18Dox_MSO_7h_rep1 ; 53338536 ; 26468894
EdUseq_ZHBTc4_18Dox_MSO_7h_rep2 ; 22745145 ; 9971664
EdUseq_ZHBTc4_24Dox_Thy_30R30E ; 19989035 ; 14369328
EdUseq_ZHBTc4_noDox_MSO_2h_rep1 ; 19282980 ; 12064340
EdUseq_ZHBTc4_noDox_MSO_2h_rep2 ; 23180459 ; 13426695
EdUseq_ZHBTc4_noDox_MSO_4h_rep1 ; 22682241 ; 17560764
EdUseq_ZHBTc4_noDox_MSO_4h_rep2 ; 21592128 ; 13008475
EdUseq_ZHBTc4_noDox_MSO_7h_rep1 ; 16525339 ; 10310072
EdUseq_ZHBTc4_noDox_MSO_7h_rep2 ; 16469700 ; 10567708
EdUseq_ZHBTc4_noDox_MSO_7h_rep3 ; 19821644 ; 9320408
EdUseq_ZHBTc4_noDox_Thy_30R30E ; 26363601 ; 18521705
Repliseq_mBMMSC_G1 ; 16358215 ; 10597883
Repliseq_mBMMSC_S1 ; 13596317 ; 10961483
Repliseq_mBMMSC_S2 ; 14589877 ; 11721256
Repliseq_mBMMSC_S3 ; 17150300 ; 10722005
Repliseq_mBMMSC_S4 ; 20221408 ; 9189176
Repliseq_MEF_G1 ; 14481432 ; 9146035
Repliseq_MEF_S1 ; 25534321 ; 18414119
Repliseq_MEF_S2 ; 19017059 ; 14242323
Repliseq_MEF_S3 ; 22812294 ; 14310109
Repliseq_MEF_S4 ; 22831063 ; 10488820

Repliseq_mESC_Nuc_G1 ; 38691779 ; 26919122
Repliseq_mESC_Nuc_S1 ; 32673381 ; 24245898
Repliseq_mESC_Nuc_S2 ; 36679797 ; 22907535
Repliseq_mESC_Nuc_S3 ; 31089196 ; 15275808
Repliseq_mESC_Nuc_S4 ; 41512082 ; 22032246
Repliseq_mESC_G1 ; 15112929 ; 11524825
Repliseq_mESC_S1 ; 16424139 ; 12929862
Repliseq_mESC_S2 ; 14388419 ; 10295360
Repliseq_mESC_S3 ; 14237837 ; 8145535
Repliseq_mESC_S4 ; 15232793 ; 9409160
EUseq_MEF_G1 ; 15935828 ; 3023850
EUseq_MEF_S1 ; 22112420 ; 4613709
EUseq_MEF_S2 ; 18604455 ; 4868283
EUseq_MEF_S3 ; 17083002 ; 2354323
EUseq_MEF_S4 ; 22375568 ; 5868589
EUseq_MEF_G2_M ; 20711414 ; 4553568
EUseq_mESC_G1_rep1 ; 14046898 ; 1412970
EUseq_mESC_G1_rep2 ; 36000297 ; 15255046
EUseq_mESC_G2_M_rep1 ; 45794629 ; 7674271
EUseq_mESC_G2_M_rep2 ; 41241524 ; 14680823
EUseq_mESC_S1_rep1 ; 25936344 ; 4842681
EUseq_mESC_S1_rep2 ; 26143987 ; 10300944
EUseq_mESC_S2_rep1 ; 15852923 ; 2987210
EUseq_mESC_S2_rep2 ; 52165792 ; 20286208
EUseq_mESC_S3_rep1 ; 14089542 ; 2620854
EUseq_mESC_S3_rep2 ; 45102034 ; 18345946
EUseq_mESC_S4_merged ; 63085757 ; 18197996
EUseq_mESC_S4_rep1 ; 25489980 ; 4806086
EUseq_mESC_S4_rep2 ; 37595777 ; 14518504

Antibodies

We did not use antibodies. Nascent DNA (EdU-seq and Repli-seq) was labeled with EdU and the EdU-labeled DNA was then linked to biotin using Click-iT chemistry.
Nascent RNA (EU-seq) was labeled with EU and the EU-labeled RNA was then linked to biotin using Clikc-iT chemistry.

Peak calling parameters

Peak calling was performed using custom codes and can be visualized using datasets and codes provided in the methods section and in https://github.com/VSDionellis/OCT4_ESC_IZ_replication.

Data quality

We used fastQC tool to assess the quality of fastq files.

Software

Our own data was downloaded from the internal server (UHTS-LIMS) of the University of Geneva genomics platform.
Public data was downloaded from ENA browser.

Flow Cytometry

Plots

Confirm that:

- [x] The axis labels state the marker and fluorochrome used (e.g. CD4-FITC).
- [x] The axis scales are clearly visible. Include numbers along axes only for bottom left plot of group (a 'group' is an analysis of identical markers).
- [x] All plots are contour plots with outliers or pseudocolor plots.
- [x] A numerical value for number of cells or percentage (with statistics) is provided.

Methodology

Sample preparation

Cells were harvested by trypsinization and fixed in 90% methanol overnight at -20° C. EdU detection was performed using the Click-it EdU Alexa Fluor 647 Flow Cytometry Assay Kit (Invitrogen Cat. No. C-10424) according to the manufacturer's instructions.
For EU quantification, asynchronous growing mESCs and MEFs were treated with EU for 30 minutes before collection by trypsin and fixed in 90% methanol/PBS. Fixed cells were processed for EU imaging following with the Click-IT RNA imaging kit (Invitrogen C10330) but using the Alexa 647 Azide antibody (Invitrogen A10277) instead. The samples were stained with FxCycle Violet Stain (F10347) and analysed in a Beckman Coulter Cytoflex device.

Instrument

Gallios, Model 2L/8C, Beckman Coulter

Software

Kaluza, version 2.1, Beckman Coulter

Cell population abundance

At least 15,000 cells were evaluated per sample.

Gating strategy	Cells were gated by FSC/SSC and FSC/TOF, then by PI peak area height/PI peak height to eliminate clumped cells (or FXCycle Violet for EU experiments), then by EdU-Alexa-647 or EU-Alexa-647 signal.

☒ Tick this box to confirm that a figure exemplifying the gating strategy is provided in the Supplementary Information.
